# In Situ Observation on Rate-Dependent Strain Localization of Thermo-Induced Shape Memory Polyurethane

**DOI:** 10.3390/polym11060982

**Published:** 2019-06-04

**Authors:** Jian Li, Qianhua Kan, Kaijuan Chen, Zhihong Liang, Guozheng Kang

**Affiliations:** 1 State Key Laboratory of Traction Power, Southwest Jiaotong University, Chengdu 610031, China; lijian12306@foxmail.com (J.L.); guozhengkang@home.swjtu.edu.cn (G.K.); 2 Applied Mechanics and Structure Safety Key Laboratory of Sichuan Province, School of Mechanics and Engineering, Southwest Jiaotong University, Chengdu 610031, China; kaijuanchen@my.swjtu.edu.cn (K.C.); zhihongliang126@163.com (Z.L.)

**Keywords:** shape memory polyurethane, rate-dependence, strain localization, internal heat generation, thermo–mechanical coupling

## Abstract

In situ monotonic tensile experiments of thermo-induced shape memory polyurethane (SMPU) at different loading rates were carried out by the digital image correlation (DIC) method and infrared camera FLIR^®^-A655sc in natural convection (NC) and forced convection (FC) conditions, respectively. The multiform strain localization of SMPU was observed by the DIC method, and the influence of thermo–mechanical coupling on the strain localization was analyzed by using the FLIR to measure the temperature field caused by the internal heat generation. The experimental results show that the strain localization mode strongly depends on the strain rate and convection condition, and the strain localization mode can be transformed by changing the convection condition from NC to FC. The competition mechanism between the strain hardening induced by the increasing loading rate and strain softening induced by the internal heat generation is indicated, the transition modes of strain localization are clarified, and the influences of thermo–mechanical coupling on shape memory effect are discussed.

## 1. Introduction

Thermo-induced shape memory polyurethane (SMPU) is one kind of thermo-sensitive polymer, and the change of its Young modulus in the vicinity of glass transition temperature ranges from two to three orders of magnitude [1,2,3]. In the tensile process of SMPU, the elastic deformation occurs firstly, and a rapid stress drop appears after yielding (called strain softening), and subsequent loading results in a strain hardening behavior due to the orientation and arrangement of molecular chains [1,3]. The reason of strain localization can be attributed to the strain softening [4,5]. When SMPU suffers from an external mechanical loading, its deformation feature presents the nonlinearity, nonequilibrium and thermo–mechanical coupling due to the large deformation and internal heat generation. In the tensile process, the nucleation and propagation of strain localization can be observed remarkably after yielding. Besides, the temperature change induced by the internal heat generation and temperature-sensitive mechanical properties of SMPU lead to a strong thermo–mechanical coupling effect during the tensile deformation, which intensifies the complexity of strain localization of SMPU. Therefore, it is necessary to further investigate the strain localization of SMPU.

Experimental investigations on the strain localization of polymers have attracted much attention. For instance, Wu and Giessen [6] introduced initial imperfections into the shear strength for describing the strain softening after yielding, and considered the orientation of molecular chains to describe the strain hardening, and thus the propagation of strain localization could be reproduced. Lanzoni and Tarantino [7] thought that the local damage induced the nucleation of strain localization, and a two-phase model, including a perfect phase and a damaged phase, was proposed to describe the propagation of strain localization. It was found that the intrinsic strain softening of SMPU was a sufficient condition of strain localization, the subsequent strain hardening could restrain the continuous development of local large deformation (necking) and promote the propagation of strain localization [4,6,8]. Li and Buckley [9] found the intrinsic anisotropy (i.e., initial pre-orientation of molecular chains) could retard the nucleation of strain localization. 

With the development of measurement technology, in situ observations on the nucleation and propagation of strain localization and the evolution of temperature field during the tensile deformation were carried out by using the digital image correlation (DIC) method and infrared camera FLIR, respectively, which improved the deep understanding of strain localization. To investigate the propagation of strain localization of high-density polyethylene, Ye et al. [10] obtained the distribution of strain field in tension by using the DIC method. From the strain contours of specimen surface, the evolutions of actual strain rates along the loading direction were obtained for characterizing the intrinsic response of strain rate and localized deformation. It was found that the actual strain rates in different positions of the specimen surface were more significant with the increasing strain rate. To improve the understanding of strain localization, the transition mode of strain localization of polymers was also investigated in detail. Jiang et al. [11] found that the influence of ambient temperature on the ductile–brittle transition in the tension of polypropylenes was remarkable. Therefore, the transition between the crazing yielding and shear yielding could be restrained by the temperature change. When the temperature increases, the shear yielding plays a dominant role; on the contrary, the crazing yielding could play a dominant role, i.e., the competition between the crazing yielding and shear yielding dominates the subsequent fracture mode. Zhang et al. [12] also observed the ductile–brittle transition in the scratching by using the FLIR. Subsequently, Jiang et al. [13] simulated the transition by using finite element code ABAQUS. Recently, Zhang et al. [14] carried out tensile experiments of the annealed and quenched poly(ethylene terephthalate)glycol and found that the aging process could regulate the transition of strain localization, that is, the annealed polymer showed a shear yielding but the quenched polymer showed a necking. It can be concluded that the temperature (including ambient temperature and temperature change induced by the internal heat generation) has a great influence on the transition of the strain localization of polymers.

Recently, the localized temperature change was observed during the tensile deformation of polymers by an infrared camera, and found that the temperature change increased with the increasing loading rate [1,15,16,17,18,19]. However, investigations on the influence of localized temperature change on the strain localization were insufficient so far. Tensile experiments of SMPU at different loading rates were carried out by Pieczyska et al. [1], it was found the remarkable temperature rise induced by the strain localization could affect the mechanical and shape memory properties. According to the localized temperature rise, the nucleation and propagation of strain localization could be deduced [17,18], and thus the influence of the thermo–mechanical coupling effect on the nucleation and propagation of strain localization should be discussed further. Besides, in the process of thermo–mechanical coupling, it was found that the temperature change strongly depended on the heat exchange with ambient temperature [3,20].

To study the correlation of strain localization and internal heat generation, tensile experiments at different loading rates were carried out in natural convection (NC) and forced convection (FC) conditions, respectively. The temperature and strain fields were obtained by using the DIC and FLIR, respectively. According to a series of monotonic tensile experiments at different loading rates in the NC condition, the influences of thermo–mechanical coupling effect on strain localization were investigated. Then, tensile experiments at different loading rates in the FC condition were performed. Comparing experimental results in the NC and FC conditions, the mechanisms of nucleation and propagation of strain localization were revealed. 

## 2. Experimental Procedure 

### 2.1. Experimental Materials

**Figure 1:**
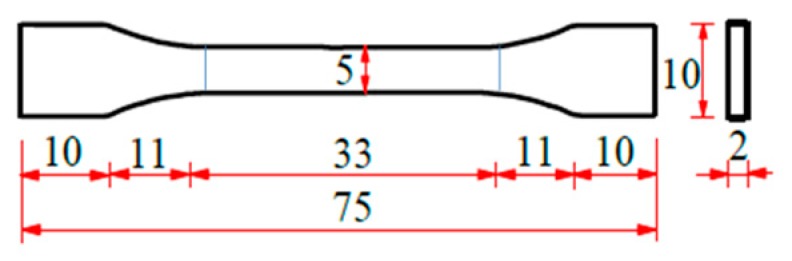
Schematic of the specimen (unit: mm) [3].

**Figure 2:**
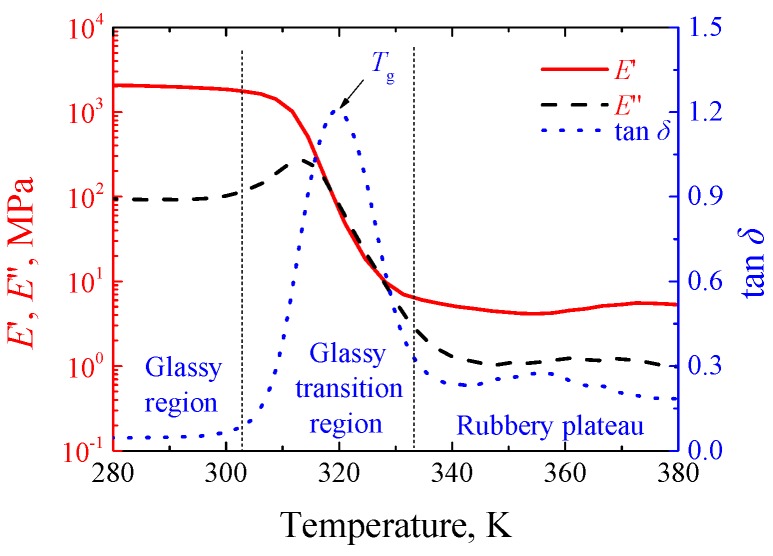
Dynamic mechanical analysis (DMA) curves of shape memory polyurethane (SMPU) (*E*′, *E*″ and tan*δ* denote the storage modulus, loss modulus and loss angle, respectively).

The experimental material was SMPU, numbered as MM4520. The material was purchased from SMP Technologies Inc. in Tokyo, Japan and exhibited shape memory effect similar to that in Reference [2]. Dumbbell specimens were injection molded with the gauge length of 33 mm [3], as shown in Figure 1. The specimens were prepared in a dry environment for experiments. The glass transition temperature of SMPU was obtained by a dynamic mechanical analysis (DMA). The DMA was conducted by a machine of DMA-Q800 (TA^®^ Instruments, New Castle, DE, USA) in a tensile mode with a temperature rate of 2 K/min and a frequency of 1 Hz from 280 K to 380 K. It can be seen from Figure 2 that the glass transition region ranges from 303 K to 333 K and the peak of tan*δ* approximates 318 K, the storage modulus changes three orders of magnitude in the narrow glass transition region, and SMPU presents a glassy state and a rubbery state below 300 K and above 340 K, respectively. From the storage modulus shown in Figure 2, stable glassy and rubbery plateaus at temperatures below 300 K and above 340 K can be observed clearly, respectively. 

### 2.2. Experimental Conditions

Tensile experiments at different temperatures were carried out by using the MTS^®^ 858-Bionix machine (MTS Systems Corp., Eden Prairie, MN, USA) to obtain basic mechanical properties of SMPU, the temperature was controlled by a SDH4004 chamber (Chongqing Inborn Experiment Instrument Ltd., Chongqing, China), whose available temperature ranged from 233 K to 423 K. Evolutions of temperature and strain fields were observed by using FLIR^®^-A655sc (FLIR^®^ system AB Inc., Waltham, MA, USA) and ARAMIS^TM^ 5M DIC (GOM^®^ mhH Ltd., Braunschweig, Germany). The FLIR-A655sc camera was placed at the front of the specimen and the DIC camera was placed at the back of the specimen. The pictures from the DIC method and FLIR camera were captured synchronously for the same specimen. Experimental conditions included the NC and FC conditions at 300 ± 0.5 K. The FC condition was performed by using double turbo fans (NMB, BL4447-04W-B49, Minebea-Matsushita Motor Corporation, Tokyo, Japan) with a rotational speed of 5500 ± 250 RPM. Both fans had the same power of 19.2 W and same direct current (DC) voltage of 12V, and they were placed at the right front and left back of the specimen, respectively, so that the decreasing temperature was uniform and the additional bending loading could be neglected. Fans were often used to simulate the intensive forced convection condition in other research fields, such as solidification behavior [21,22] and electronic components [23]. The loading rates were 0.1, 1, 5 and 10 mm/s in the NC and FC conditions, respectively. The loading displacement of all specimens was 33 mm. The loading rate represents the crosshead separation rate and the measured displacement is the crosshead displacement of the MTS^®^ 858-Bionix machine.

## 3. Experimental Results 

### 3.1. Force–Displacement Curves at Different Temperatures and Loading Rates

**Figure 3:**
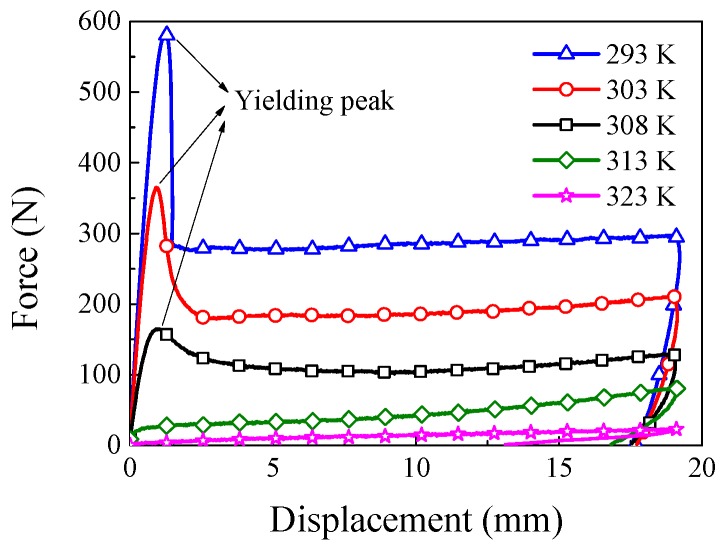
Curves of force versus displacement of SMPU at different temperatures.

The force–displacement curves at different temperatures with the loading rate of 1 mm/s are shown in Figure 3. It can be seen from Figure 3 that the influence of temperature on mechanical properties of SMPU is obvious, and the yielding peak decreased gradually and vanished finally with the increasing temperature. Since the loss modulus appeared at a peak value near 313 K, as shown in Figure 2, the viscosity was so strong that the unloading curve exhibited a strong nonlinearity. The force response decreased remarkably and the yielding peak disappeared at 323 K since SMPU presented a rubbery state at high temperature [24]. 

**Figure 4:**
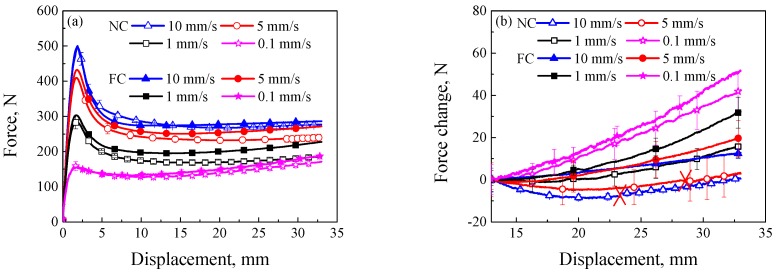
(**a**) Curves of force and displacement at different loading rates; (**b**) curves of force change and displacement at the plastic flow stage at different loading rates (symbol ‘×’ denotes the fracture of specimens at the loading displacement).

The average rate-dependent tensile experimental results with error bars in the NC and FC conditions are shown in Figure 4. Three specimens were repeated for the loading rate of 0.1 mm/s in the NC and FC conditions, respectively, and four specimens for other loading conditions. It is shown from Figure 4a that the yielding peak and force response after yielding increased with the increasing loading rate. Comparing experimental results in the NC and FC conditions, the influence of FC condition on the elastic deformation could be neglected, but its influences on the yielding peak and plastic flow stage after yielding were remarkable. To better study the plastic flow at different loading rates, the force change in the plastic flow stage was computed, as shown in Figure 4b. Since the plastic flow stage begins after the loading displacement of 13 mm, the force change is defined as the difference between the force after the loading displacement of 13 mm and that at the loading displacement of 13 mm. It was found that the force change in the NC condition decreased with the increasing loading rate. Besides, the force change decreased with the increasing loading displacement at the loading rates of 5 and 10 mm/s (called strain softening). The force change in the FC condition also decreased with the increasing loading rate, but increased with the increasing loading displacement (called strain hardening). According to different loading conditions, the force change of plastic flow stage in the FC condition was more obvious than that in the NC condition. At the loading rates of 5 and 10 mm/s, the plastic flow changed from strain softening in the NC condition to strain hardening in the FC condition. It indicates that strain softening and strain hardening at the plastic flow stage can be changed by the convection condition. It must be noted that the correlation between the force change and loading rate was still unchanged, that is, the force change decreased with the increasing loading rate.

### 3.2. Strain Field Distribution at Different Loading Rates and Convection Conditions

**Figure 5:**
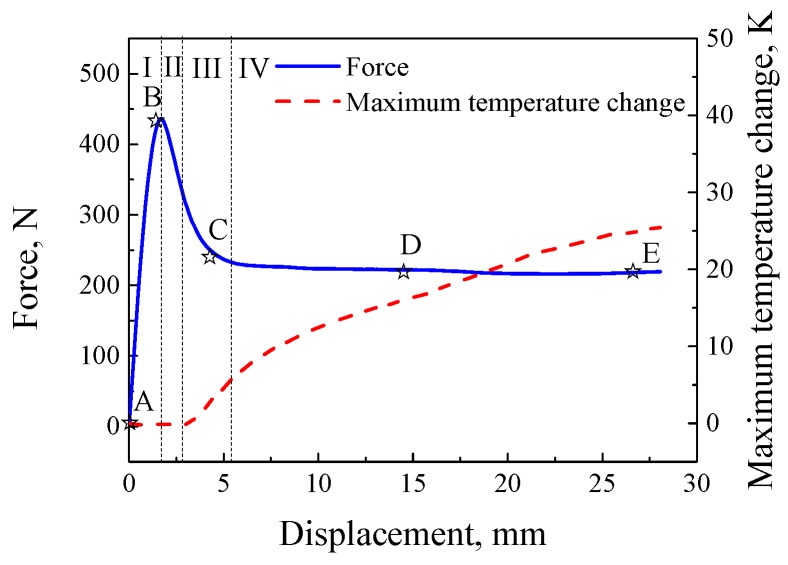
Illustration of loading partition.

Referring to the method of deformation decomposition proposed by Muhammad and Jar [25], and combining force–displacement curves and temperature change–displacement curves, the loading process can be divided into four parts by three characteristic strains, i.e., the yielding strain, nucleation strain of strain localization and flow strain. The four parts are shown in Figure 5, i.e., I: Elastic deformation, II: Strain softening (from the peak stress to the start of temperature rise), III: Nucleation of localized deformation (from the start of temperature rise to the end of strain softening) and IV: Propagation of localized deformation (from the end of strain softening to the end of loading). Correspondingly, five characteristic points are selected as point A, point B, point C, point D and point E to observe the deformation process, and they present the initial point, peak stress point, end point of strain softening, the middle point of localized propagation and the end point of loading, respectively. 

**Figure 6:**
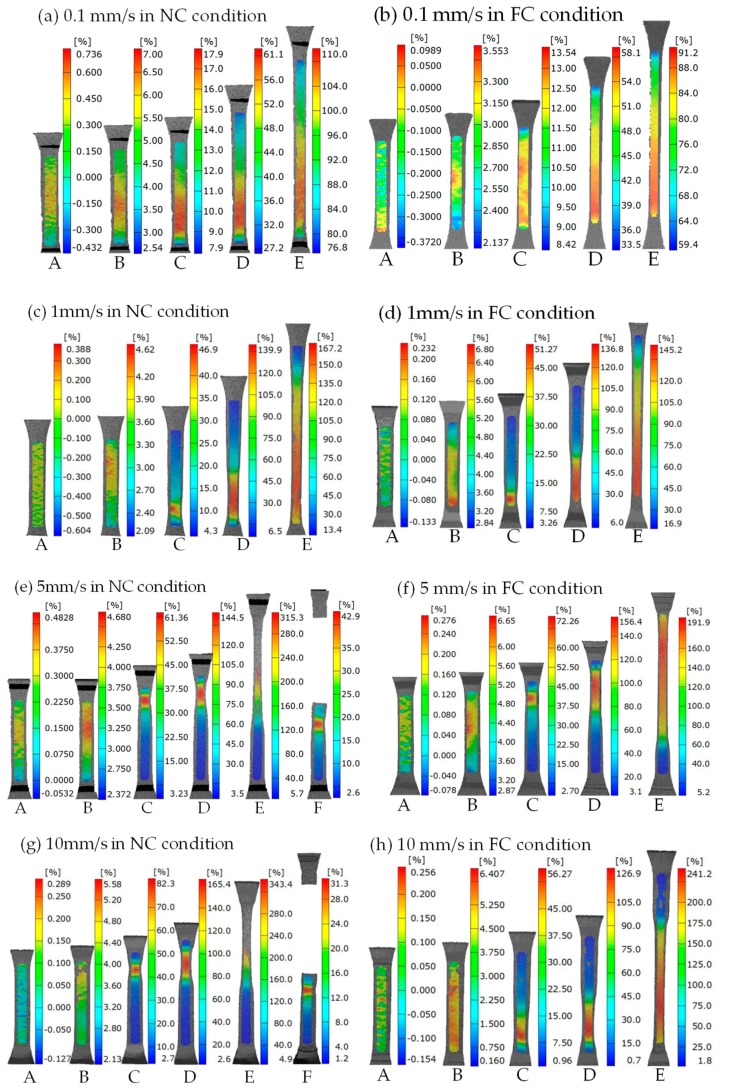
Strain contours at the loading rates of (**a**) 0.1 mm/s in the natural convection (NC) condition; (**b**) 0.1 mm/s in the forced convection (FC) condition; (**c**) 1 mm/s in the NC condition; (**d**) 1 mm/s in the FC condition; (**e**) 5 mm/s in the NC condition; (**f**) 5 mm/s in the FC condition; (**g**) 10 mm/s in the NC condition and (**h**) 10 mm/s in the FC condition. (Point F in Figure 6e,g denotes the fracture of the specimen).

The strain contours of SMPU at different loading rates in the NC and FC conditions are given in Figure 6. (To enable the reader to observe more intuitively, three typical videos of strain localizations are presented in the Supplementary Materials.). The deformation process can be obtained directly according to five characteristic points shown in Figure 5. Overall, whether it is the NC condition or FC condition, the deformation presented an obvious strain localization, and the strain localization continued propagating with the increasing displacement. The initial position of strain localization nucleated near the transition section of the specimen. The difference in the propagation mode of strain localization could be observed at different loading rates. It was seen that the maximum strain increased with the increasing loading rate, i.e., 110.0% for 0.1 mm/s, 167.2% for 1 mm/s, 315.3% for 5 mm/s and 343.4% for 10 mm/s in the NC condition, 91.2% for 0.1 mm/s, 145.2% for 1 mm/s, 191.9% for 5 mm/s and 241.2% for 10 mm/s in the FC condition, respectively. The minimum strain decreased with the increasing loading rate, i.e., 76.8% for 0.1 mm/s, 13.4% for 1 mm/s, 5.7% for 5 mm/s and 4.9% for 10 mm/s in the NC condition, 59.4% for 0.1 mm/s, 16.9% for 1 mm/s, 5.2% for 5 mm/s and 1.8% for 10 mm/s in the FC condition, respectively. The region of strain localization also decreased with the increasing loading rate, which indicates that the strain localization was more remarkable with the increasing loading rate. Comparing Figure 6a,c,e,g and Figure 6b,d,f,h, the maximum strain in the FC condition was less than that in the NC condition at a same loading rate. It is noted that the specimen fractured due to a large localized deformation, i.e., the measured maximum strain was 315.3% and 343.4%, as shown in Figure 6e,g, respectively. 

**Figure 7:**
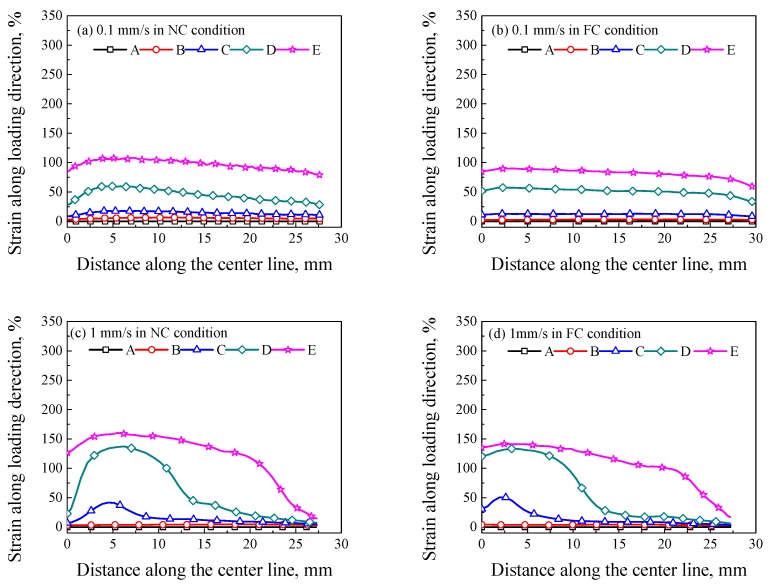

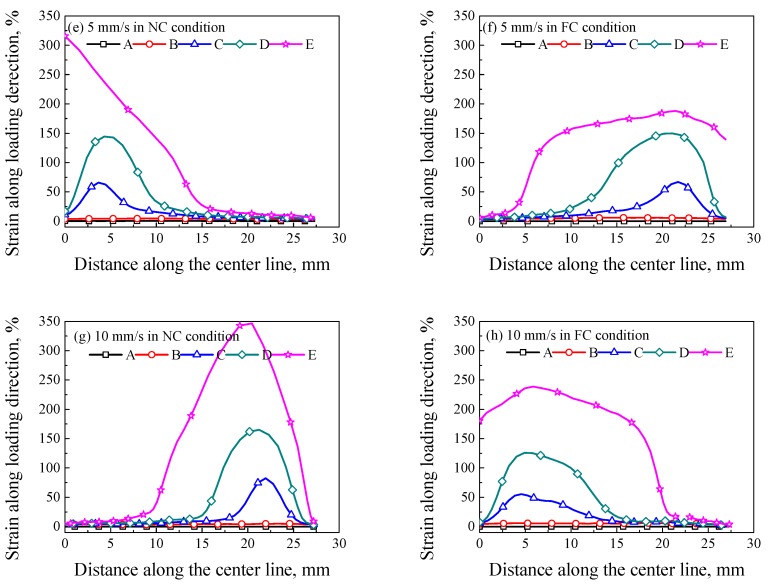
Strains along the center line of specimens at different loading rates of (**a**) 0.1 mm/s in the NC condition; (**b**) 0.1 mm/s in the FC condition; (**c**) 1 mm/s in the NC condition; (**d**) 1 mm/s in the FC condition; (**e**) 5 mm/s in the NC condition; (**f**) 5 mm/s in the FC condition; (**g**) 10 mm/s in the NC condition and (**h**) 10 mm/s in the FC condition.

The axial strain along the center line of specimens in Figure 6 is extracted, as shown in Figure 7. In Figure 7a, no obvious strain localization was observed at the loading rate of 0.1 mm/s in the NC condition, i.e., the strain along the gauge section of the specimen was approximately uniform, the deformation presented a uniform elongation mode with a weak localized deformation at point D. Moreover, the deformation in Figure 7b was more uniform than that in Figure 7a. In Figure 7c, an obvious strain localization can be observed at point C, which indicates that the strain localization nucleated from the strain softening (point C corresponds to the strain softening, as shown in Figure 5). As the strain localization increased after point D, the strain localization began to propagate, i.e., the strain localization presented a propagation mode, and the width of deformation region was enlarged from point D to point E. In Figure 7e,g, the plastic flow was accompanied by a sharp peak strain and the peak strain increased with the increasing loading displacement, i.e., the strain localization presented a localized deformation mode. It can be concluded that, the strain localization mode can transform from a uniform elongation mode (loading rate of 0.1 mm/s) to a propagation mode (loading rate of 1 mm/s), and then to a localized deformation mode (loading rate of 10 mm/s) with the increasing loading rate in the NC condition. In Figure 7d, the peak strain increased with the increasing displacement in the FC condition from point C to point D, but the peak strain only extended without significant increase after point D, which presented a propagation mode of strain localization. In Figure 7f, the peak strain extended after point D without a sharp peak strain, which is similar with that in Figure 7c,d rather than that in Figure 7e. In Figure 7h, the peak strain extended and increased after point D, which presented a mixed mode of strain localization and strain propagation. To sum up, the mode of strain localization could be transformed by the increasing loading rate and changing convection condition from NC to FC.

### 3.3. Temperature Field Distribution at Different Loading Rates and Convection Conditions

**Figure 8:**
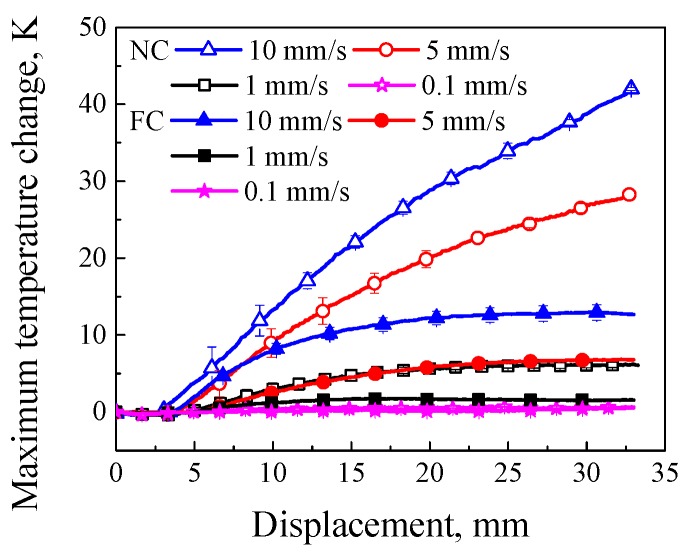
Curves of maximum temperature change and displacement at different loading rates in the NC and FC conditions.

The temperature field can be monitored synchronously by using the FLIR camera during the tensile deformation. The correlations between the maximum temperature change (the current maximum temperature subtract the initial temperature) with error bars and loading displacement are shown in Figure 8. It was found that the maximum temperature change in the FC condition increased with the increasing loading rate, and was less than that in the NC condition at the same loading rate; in the meantime, the slop of maximum temperature change–displacement curve decreased with the increasing loading displacement, especially at a relative low loading rate and in the FC condition, which was the result of a stronger heat exchange with the surrounding environment in the FC condition than that in the NC condition. 

**Figure 9:**
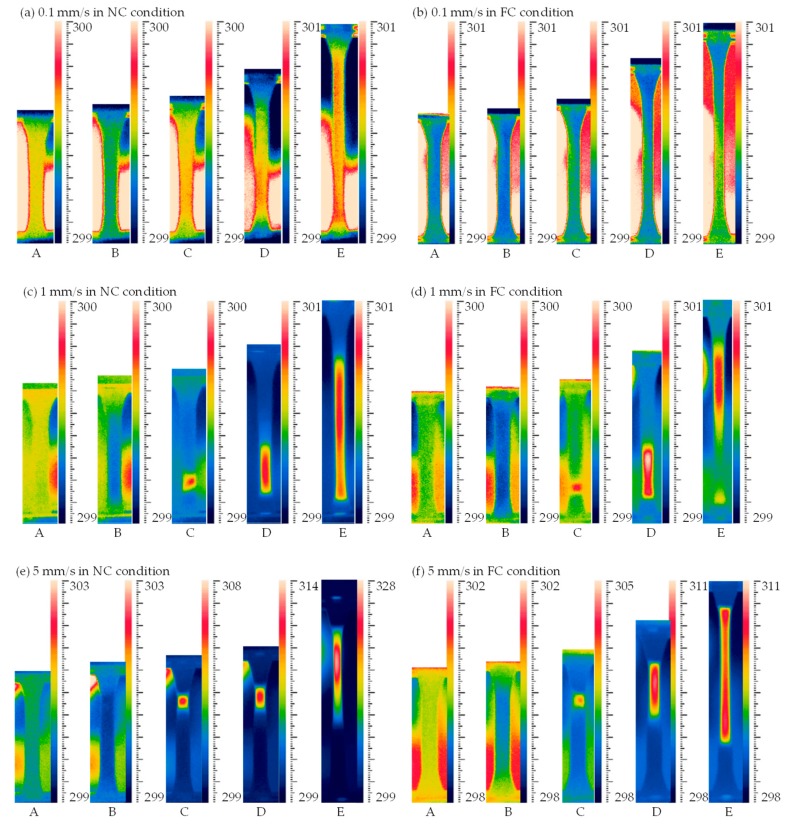

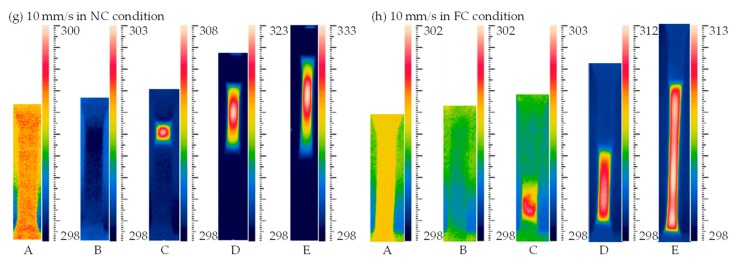
Temperature contours at different loading rates of (**a**) 0.1 mm/s in the NC condition; (**b**) 0.1 mm/s in the FC condition; (**c**) 1 mm/s in the NC condition; (**d**) 1 mm/s in the FC condition; (**e**) 5 mm/s in the NC condition; (**f**) 5 mm/s in the FC condition; (**g**) 10 mm/s in the NC condition and (**h**) 10 mm/s in FC condition.

Temperature field contours at different loading rates are shown in Figure 9. The picture at point A is the undeformed reference state. It was found that the temperature of point B in all figures was less than that of point A since the elastic deformation decreased the temperature of specimen [1,3]. The following plastic deformation generated the internal heat and resulted in a temperature rise of the specimen, and the temperature rise further promoted the plastic flow, i.e., thermo–mechanical coupling effect [3,18]. In Figure 9a,b, temperature fields present approximately uniform distributions at the loading rate of 0.1 mm/s in the NC and FC conditions; however, temperature fields in Figure 9c–h present obvious temperature localizations at the loading rates of 1, 5 and 10 mm/s in the NC and FC conditions. The temperature rise was generated from a local region and then propagated to its adjacent region with the increasing loading displacement. It is seen from Figure 9a–h that the region of obvious temperature change on specimen surface became smaller and smaller with the increasing loading rate, while the maximum temperature rise increased with the increasing loading rate. In addition, the temperature rise in the FC condition was less than that in the NC condition, and its distribution in the FC condition was wider than that in the NC condition. That is, the convection condition affected the value of temperature change and its distribution remarkably, which is similar with its effects on the strain distribution discussed in Section 3.2.

**Figure 10:**
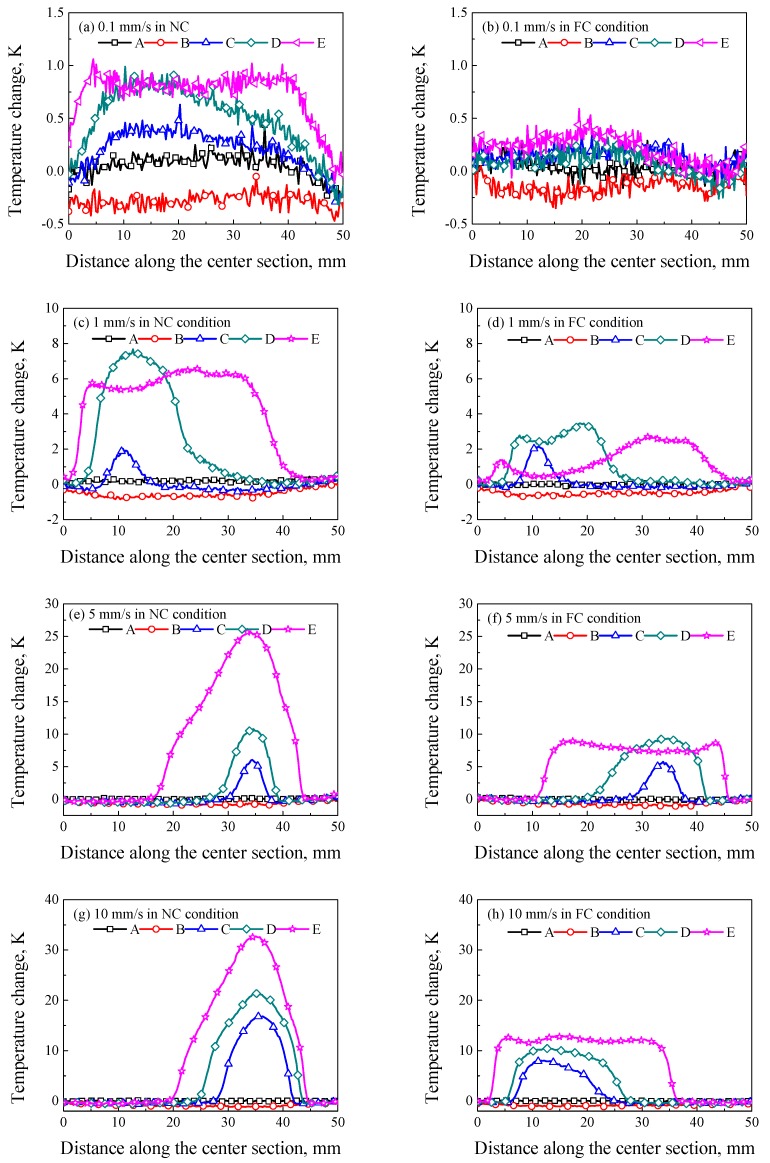
Temperature changes along the center section of specimens at different loading rates of (**a**) 0.1 mm/s in the NC condition; (**b**) 0.1 mm/s in the FC condition; (**c**) 1 mm/s in the NC condition; (**d**) 1 mm/s in the FC condition; (**e**) 5 mm/s in the NC condition; (**f**) 5 mm/s in the FC condition; (**g**) 10 mm/s in the NC condition and (**h**) 10 mm/s in FC condition.

The temperature changes along the center line of specimens are shown in Figure 10. It is seen that the temperature change at point A decreased due to the thermo–elastic effect. However, the plastic deformation increased the temperature rise at a local position, and the maximum temperature rise at different loading rates ranged from 1 K to 33 K. In Figure 10a, the temperature change at the loading rate of 0.1 mm/s was low, and its maximum value was only about 1 K since the internal heat generation depended on the loading rate and heat exchange rate. In Figure 10b, the temperature change was close to zero since the FC enhanced the heat exchange. In Figure 10c–h, the local plastic deformation induced the local temperature rise in the initial stage of strain localization. The stress concentration induced by the initial defects led to the disentanglement of molecular chains, and the friction between molecular chains generated a local temperature rise, which is the origin of strain localization nucleation [6,7,26]. In Figure 10c, the temperature change propagated from a local position to a wide region. In Figure 10e,g, the temperature changes continued to increase to maximum values of 26 K and 33 K at a local region, respectively. The temperature change increased but its width decreased with the increasing loading rate in the NC condition. In Figure 10d, the temperature rise propagated from a local region to a wide region with a relative stable temperature change, and the temperature decreased at the region after deformation due to the FC, as shown at point E. In Figure 10f,h, the temperature change propagated stably from a local region to a wide region with a relatively high temperature change. 

### 3.4. The Correlation of Strain Localization and Temperature Change

**Figure 11:**
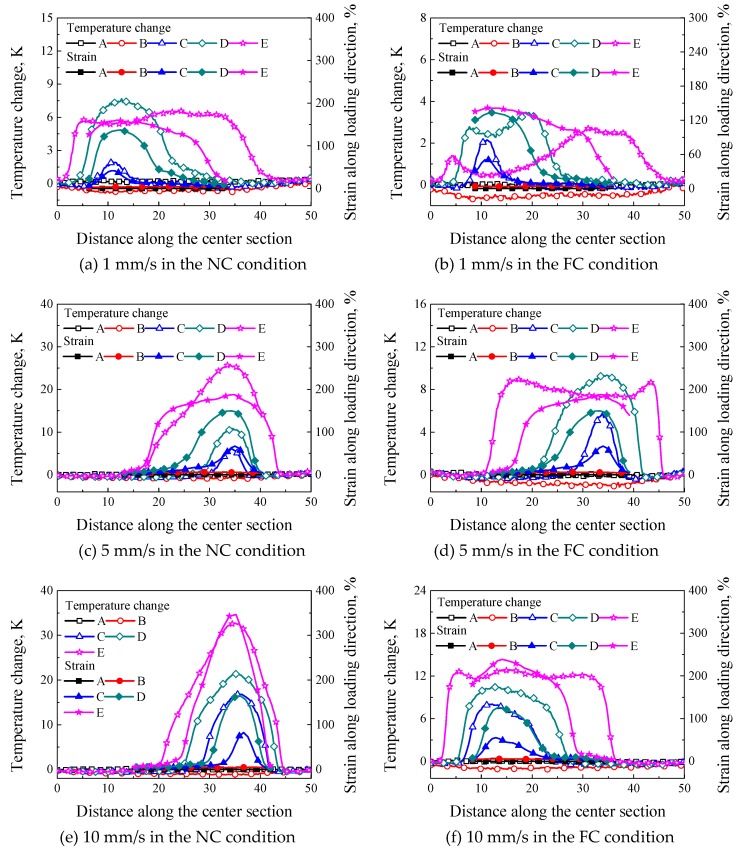
The correlation between the temperature change and strain at different loading rates and convection conditions: (**a**) 1 mm/s in the NC condition; (**b**) 1 mm/s in the FC condition; (**c**) 5 mm/s in the NC condition; (**d**) 5 mm/s in the FC condition; (**e**) 10 mm/s in the NC condition and (**f**) 10 mm/s in the FC condition.

To understand the correlation of strain localization and temperature change, the strain and temperature distributions of the same specimen were compared, as shown in Figure 11a–f. It was seen that the distribution regions of local deformation were similar with those of temperature change at different loading rates, especially that in the NC condition. When the strain localization nucleated from a local region, the temperature localization occurred at the same position, i.e., the temperature distribution changed with the propagation of strain localization. The strain distributions along the center section in the NC condition shown in Figure 11a,c,e were more similar with temperature distribution than those in the FC condition shown in Figure 11b,d,f since the FC changed the heat exchange. For instance, as shown at point E in Figure 11b, the maximum strain distribution was located at the bottom of the specimen, while the maximum temperature was located at the upper part of the specimen. The reason is that the strain at point D in Figure 11b was up to 136.8%, which was close to 145.2% at point E in Figure 11b, i.e., during the deformation from point D to point E, the strain at the bottom of the specimen hardly increased with the increasing loading displacement, but the temperature change decreased obviously due to the heat exchange (see Figure 11b). As a result, the temperature change at the upper part of the specimen increased due to the continuously increasing deformation at point E. A similar phenomenon is observed in Figure 11d. Therefore, the effect of temperature change induced by the internal heat generation on the strain localization can be changed. In addition, it can be seen from Figure 11a,c that the maximum temperature change at the loading rate of 1 mm/s in the NC condition was far less than that at the loading rate of 5 mm/s in the NC condition, but it was similar to that at the loading rate of 5 mm/s in the FC condition shown in Figure 11d, which indicates the temperature change due to the internal heat generation can be changed by the FC condition.

## 4. Discussion

To indicate the thermo–mechanical coupling effect of thermo-induced SMPU, the discussion of the rate-dependent strain localization and internal heat generation is very important. It is noted from Figure 2 and Figure 3 that mechanical properties of SMPU are more sensitive to the temperature than that of common thermo-plastic polymers, e.g., the magnitude of storage modulus ranges from 1 to 850 MPa in Reference [14] is less than that from 5 to 2000 MPa in present work, and common thermo-plastic polymers do not exhibit a stable rubbery plateau [14]. Therefore, the internal heat generation of SMPU induced by the plastic dissipation has a stronger effect on the strain localization than common thermo-plastic polymers. In the meantime, the temperature change on the surface of the specimen depends on the loading rate and convection mode due to the different heat exchange rates. As a result, the rate-dependent strain localization can further affect the internal heat generation. In situ observations on rate-dependent tensile experiments of SMPU in the present work can enhance the understanding of correlation between the strain localization and internal heat generation.

Stress–strain response of common glassy polymers exhibits an obvious rate-dependence [27,28], that is, the stress response increases with the increasing loading rate. However, a higher temperature change of SMPU due to the internal heat generation can change the rate-dependence since the higher temperature change can induce the more remarkable thermo-softening behavior. It was found from Figure 8, Figure 9 and Figure 10 that, the temperature change increased with the increasing loading rate, promoted the transition from a glassy state to a rubbery state and degraded the mechanical properties of SMPU. It can be used to explain why the force change at loading rates of 5 and 10 mm/s in the NC condition decreased with the increasing loading displacement at the initial plastic flow stage shown in Figure 4, i.e., the thermal softening plays a dominant role in the NC condition. It was also found that the force change at the plastic flow stage in the FC condition was larger than that in the NC condition in Figure 4. The reason is that most of the temperature change was taken away by the heat exchange in the FC condition. The force change presented its intrinsic rate-dependence, that is, the mechanical response increased with the increasing loading rate, and the rate-dependent strain hardening played a dominant role in the FC condition. There is a competition between strain rate-hardening and thermo-softening in the thermo–mechanical coupling process. Therefore, the rate-dependent stress–strain curve of SMPU in tension is not an intrinsic mechanical response due to the thermo–mechanical coupling effect, as a result, only force–displacement curves are provided in the work. 

SMPU is different from common polymers due to its shape memory effect. Shape memory effect can be characterized by the strain recovery under zero stress (free recovery) and stress recovery under fixed strain (constrained recovery) [29,30,31]. In the constrained recovery process, the recovery stress firstly increases with the increasing temperature, and then decreases with the increasing temperature due to the stress relaxation at high temperature [30,31]. At the plastic flow stage, the temperature change increases with the increasing displacement, the shape memory effect can be activated at a high temperature. Therefore, the stress induced by the temperature at the plastic flow stage consists of three parts, i.e., stress induced by the decreasing modulus, stress induced by shape memory effect and relaxed stress. As a result, the influence of shape memory effect on the strain localization is difficult to be discussed separately. 

**Figure 12:**
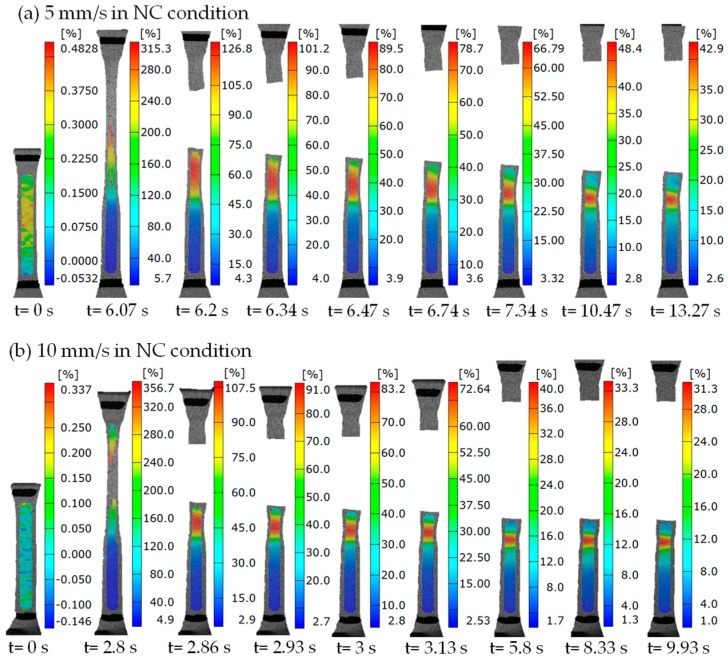
Pictures of strain contours from tension to fracture at different moments with the loading rates of (**a**) 5 mm/s and (**b**) 10 mm/s in the NC condition.

**Figure 13:**
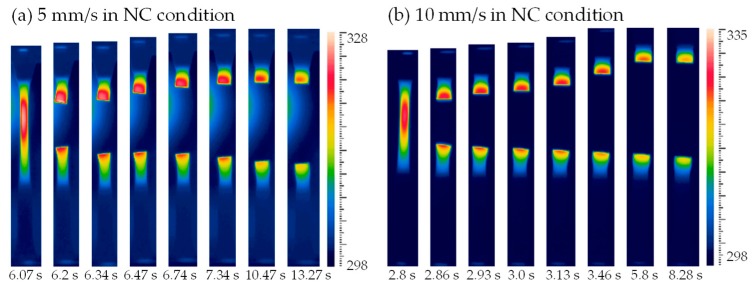
Temperature contours after fracture at different moments at the loading rates of (**a**) 5 mm/s and (**b**) 10 mm/s in the NC condition.

**Figure 14:**
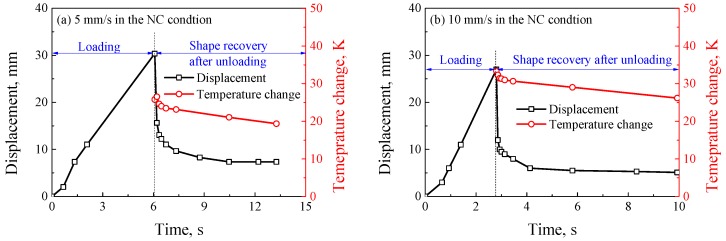
The curves of displacement and temperature change with time at the loading rates of (**a**) 5 mm/s and (**b**) 10 mm/s in the NC condition.

However, it is found from Figure 12 that the local large deformation could be recovered after the specimen fractures, and the unrecoverable strain at the loading rate of 5 mm/s was larger than that at the loading rate of 10 mm/s in the NC condition, it implies the shape memory effect can be activated by the high heat generation, and the recovery capacity of shape memory depends on the loading rate. The temperature contours at different moments after the specimen fractures were captured, as shown in Figure 13. It was seen that the temperature rise decreased gradually, but it was always much higher than the ambient temperature. Comparing Figure 13a,b, it was found that the temperature rise at the loading rate of 5 mm/s was lower than that at the loading rate of 10 mm/s due to a longer time to conduct heat exchange at a lower loading rate. To quantitatively indicate the relationship between the shape recovery and temperature rise, the curves of displacement and temperature change with time at the loading rates of 5 mm/s and 10 mm/s in the NC condition are shown in Figure 14. It was found that the curves of the displacement recovery with time after fracture at loading rates of 5 mm/s and 10 mm/s in the NC condition were similar, when the temperature change decreased with the increasing fracture time, the recovery rate of displacement approximated to zero. The reason is that once the temperature drops to below glass transition temperature, the shape memory effect of SMPU can be neglected. In the meantime, the residual strain can be observed due to the large localized deformation [29,32].

## 5. Conclusions

In situ observations on monotonic tensile experiments at different loading rates were carried out in the NC and FC conditions by using the DIC and FLIR methods, respectively, the influences of loading rate and convection condition on the strain localization were investigated. The main conclusions are as following:

(1) The thermo–mechanical coupling effect was investigated by the rate-dependent experiments. The temperature distribution was similar with strain distribution in the NC condition, and the similarity can be changed by the FC condition. 

(2) The strain localization mode can be changed from a uniform elongation mode to a propagation mode, and to a local large deformation mode with the increasing loading rate. In the meantime, the strain localization can be changed from a local large deformation mode to a propagation mode by the FC condition.

(3) The competition between rate-dependent strain hardening and temperature-induced strain softening occurs at the plastic flow stage. When the temperature change is relatively large, the temperature-induced strain softening plays a dominant role; on the contrary, the rate-dependent strain hardening plays a dominant role. 

(4) The shape memory effect can be activated by the internal heat generation at a relative high loading rate and the deformation after fracture can be recovered partly.

## Supplementary Materials

The following are available online at https://www.mdpi.com/2073-4360/11/6/982/s1, Video S1: the strain localization of SMPU with the loading rate of 1 mm/s in the NC condition; Video S2: the strain localization of SMPU with the loading rate of 10 mm/s in the NC condition; Video S3: the strain localization of SMPU with the loading rate of 10 mm/s in the FC condition.
